# Evidence for classification of c.1852_1853AA>GC in MLH1 as a neutral variant for Lynch syndrome

**DOI:** 10.1186/1471-2350-12-12

**Published:** 2011-01-19

**Authors:** Adela Castillejo, Carla Guarinos, Ana Martinez-Canto, Victor-Manuel Barbera, Cecilia Egoavil, Maria-Isabel Castillejo, Lucia Perez-Carbonell, Ana-Beatriz Sanchez-Heras, Angel Segura, Enrique Ochoa, Rafael Lazaro, Clara Ruiz-Ponte, Luis Bujanda, Montserrat Andreu, Antoni Castells, Angel Carracedo, Xavier Llor, Juan Clofent, Cristina Alenda, Artemio Paya, Rodrigo Jover, Jose-Luis Soto

**Affiliations:** 1 Laboratorio Investigación. Hospital Universitario Elche. Elche. Spain; 2 Laboratorio Investigación. Hospital Universitario Alicante. Alicante. Spain; 3 Unidad Consejo Genético Cáncer. Hospital Universitario Elche. Elche. Spain; 4 Unidad Consejo Genético Cáncer. Hospital Universitario La Fe. Valencia. Spain; 5 Unidad Biopatología Molecular. Hospital Provincial Castellón. Castellón. Spain; 6 Servicio Anatomía Patológica. Hospital La Plana Castellón. Castellón. Spain; 7 Fundación Pública Galega de Medicina Xenómica, CIBERER, Grupo de Medicina Xenómica-Universidad de Santiago de Compostela. Santiago de Compostela. Spain; 8 Hospital Donostia. Instituto Biodonostia. Universidad del País Vasco. CIBERehd. San Sebastián. Spain; 9 Departamento de Gastroenterología. Hospital del Mar. Barcelona. Spain; 10 Departmento de Gastroenterología. Institut de Malaties Digestives, Hospital Clínic, IDIBAPS, CIBERehd, Universidad de Barcelona. Barcelona. Spain; 11 Department of Medicine and Cancer Center. University of Illinois at Chicago. Chicago, Il. USA; 12 Servicio de Medicina Digestiva. Hospital Universitario La Fe. Valencia. Spain; 13 Servicio de Anatomía Patológica. Hospital Universitario Alicante. Alicante. Spain; 14 Unidad de Gastroenterología. Hospital Universitario Alicante. Alicante. Spain

## Abstract

**Background:**

Lynch syndrome (LS) is an autosomal dominant inherited cancer syndrome characterized by early onset cancers of the colorectum, endometrium and other tumours. A significant proportion of DNA variants in LS patients are unclassified. Reports on the pathogenicity of the c.1852_1853AA>GC (p.Lys618Ala) variant of the *MLH1 *gene are conflicting. In this study, we provide new evidence indicating that this variant has no significant implications for LS.

**Methods:**

The following approach was used to assess the clinical significance of the p.Lys618Ala variant: frequency in a control population, case-control comparison, co-occurrence of the p.Lys618Ala variant with a pathogenic mutation, co-segregation with the disease and microsatellite instability in tumours from carriers of the variant. We genotyped p.Lys618Ala in 1034 individuals (373 sporadic colorectal cancer [CRC] patients, 250 index subjects from families suspected of having LS [revised Bethesda guidelines] and 411 controls). Three well-characterized LS families that fulfilled the Amsterdam II Criteria and consisted of members with the p.Lys618Ala variant were included to assess co-occurrence and co-segregation. A subset of colorectal tumour DNA samples from 17 patients carrying the p.Lys618Ala variant was screened for microsatellite instability using five mononucleotide markers.

**Results:**

Twenty-seven individuals were heterozygous for the p.Lys618Ala variant; nine had sporadic CRC (2.41%), seven were suspected of having hereditary CRC (2.8%) and 11 were controls (2.68%). There were no significant associations in the case-control and case-case studies. The p.Lys618Ala variant was co-existent with pathogenic mutations in two unrelated LS families. In one family, the allele distribution of the pathogenic and unclassified variant was in *trans*, in the other family the pathogenic variant was detected in the MSH6 gene and only the deleterious variant co-segregated with the disease in both families. Only two positive cases of microsatellite instability (2/17, 11.8%) were detected in tumours from p.Lys618Ala carriers, indicating that this variant does not play a role in functional inactivation of *MLH1 *in CRC patients.

**Conclusions:**

The p.Lys618Ala variant should be considered a neutral variant for LS. These findings have implications for the clinical management of CRC probands and their relatives.

## Background

Genetic testing is conducted for diverse purposes, including confirmation of diagnosis, risk prediction, carrier testing and reproductive decision-making. The identification of germ-line mutations in patients with inherited cancer syndromes enables them to be included in cancer surveillance programmes. Such programmes are effective in reducing cancer mortality in the families concerned. Moreover, family members who do not carry the mutation can be treated safely as low-risk individuals, avoiding unnecessary screening and preventing anxiety in the individuals concerned. Unfortunately, the number of suspected familial cancer cases in which a causative mutation is identified is far from ideal. All members from a family with a strong history of cancer and no causative mutation detected are included in a surveillance program. Identification of mutations depends on the specific syndrome and the criteria applied to select patients for genetic analyses.

The results of sequence-based genetic tests may be reported to physicians as: 1) positive, in which a mutation that clearly disrupts gene function is detected and is highly likely to have clinical consequences; 2) a genetic variant is detected but it is not known whether the variant has any effect on gene function that might confer an increased cancer risk (these variants are known as variants of uncertain/unclassified significance or unclassified variants [UVs]); and 3) negative, in which deleterious variant or UV is detected [1].

The majority of UVs are missense mutations or small in-frame deletions. The human gene pool harbours a vast number of rare missense substitutions, 70% of which are at least mildly deleterious [2]. Integration of various lines of evidence may help to classify UVs. Information on: 1) frequencies in cases and controls, 2) co-occurrence (in *trans*) with deleterious mutations, 3) co-segregation with disease in pedigrees, 4) pathological factors, 5) amino acid polarity or size, 6) evolutionary conservation of the residue, 7) splice predictions and 8) *in vitro *and/or *in vivo *functional assays may enable UVs to be classified as pathogenic or non-pathogenic [3].

Lynch syndrome (MIM# 120435) (LS) is an autosomal dominant inherited cancer syndrome characterized by early onset colorectal cancer (CRC), cancer of the endometrium and tumours of the stomach, pancreas, small intestine, ovary, bladder and bile duct [4]. LS-associated tumours are characterized by DNA mismatch repair (MMR) deficiency, which may be evidenced by microsatellite instability (MSI) or loss of expression of MMR proteins using immunohistochemistry [5]. The proportion of genetic UVs in LS varies from 1/5 to 1/3 of all unique variants detected [6].

The *MLH1 *p.Lys618Ala (c.1852_1853AA>GC) variant was initially considered a deleterious variant based on its recurrent presence in LS families, *in silico *predictions and *in vitro *experiments on its functional effect. However, recent data on its co-segregation with LS have cast doubt on its clinical significance [6]. In this study, we provide evidence supporting the contention that this variant has no significant implications in LS.

The following approach was used to assess the clinical significance of the p.Lys618Ala variant: frequency in a control population, case-control and case-case comparisons, co-occurrence with a pathogenic mutation, co-segregation with the disease and MSI in tumours from carrier individuals.

## Methods

### Controls and sporadic and familial CRC cases

We genotyped the p.Lys618Ala variant in *MLH1 *in 1034 individuals (373 sporadic CRC patients, 250 index subjects from families suspected of having LS [revised Bethesda Guidelines] and 411 controls). The controls were selected from the same hospitals, had no personal histories of cancer and had diagnoses unrelated to the variables of interest. They were matched for age, gender and race/ethnicity with the sporadic CRC patients.

No familial history of cancer was available from the control group. Patients diagnosed at an age over 50 years and not referred to Genetic Counselling Units were considered as sporadic CRC. Samples from sporadic CRC patients were obtained from the Elche University Hospital BioBank and the Castellon Provincial Hospital BioBank. Written consent to be included in the respective biobanks was obtained from each patient. CRC patients, as index subjects from families with suspicion of LS that attended Genetic Counselling at the Cancer Units of the Elche and La Fe Hospitals, were recruited. The study was approved by the Ethics Committee of the Elche University Hospital.

The median age of patients in the sporadic CRC group was 70 years (range, 52-93 years), 47 years (range, 21-87 years) for the familial group and 71 years (range, 25-96 years) for the controls. The sex distribution was 58% men and 42% women for the sporadic CRC group and 53.3% men and 46.7% women for the controls.

### Families carrying the p.Lys618Ala variant

Three characterized LS families that fulfilled the Amsterdam II Criteria and that consisted of members with the p.Lys618Ala variant were included to assess co-occurrence and co-segregation. Two families attended the Genetic Counselling in Cancer Units of the Elche and La Fe Hospitals and one family was a member of the EPICOLON cohort [7].

Concomitant deleterious variants were detected in two of the families: one in the *MLH1 *gene (c.676C>T; p.Arg226X) and the other in the *MSH6 *gene (c.3013C>T; p.Arg1005X). Seventeen affected and unaffected family members from these two families were tested for the pathogenic and p.Lys618Ala variants.

### Genotyping of the MLH1 p.Lys618Ala variant

DNA from blood cells (familial cancer cases and controls) or colorectal mucosa of normal appearance (sporadic cases) was used for the c.1852_1853AA>GC variant genotyping. This was assessed using the iPLEX Gold method (Sequenom, CA, USA), in which single-base extension and MALDI-TOF technology are employed for allelic discrimination. These experiments were carried out at the Centro Español de Genotipado (CEGEN) genotyping platform facilities. Quality control for genotyping was conducted by direct sequencing of familial cancer subjects who underwent genetic analysis for MLH1 (49/1034, 4.7%).

### Microsatellite instability and MLH1 immunohistochemical expression

A subset of colorectal tumour DNA samples from 17 patients carrying the p.Lys618Ala variant (eight from the familial group and nine from the sporadic CRC group) was screened for MSI status using five mononucleotide markers (BAT26, BAT25, NR21, NR24 and NR27) and multiplex PCR as previously described by Buhard et al [8].

Tumours from p.Lys618Ala carrier cases in the familial group (seven index subjects and one relative) were also analysed for MLH1 protein expression using immunohistochemistry and anti-MLH1 antibodies (PharMingen, CA, USA) as described elsewhere [7]. Tumour cells were judged negative for protein expression only if they lacked staining in a sample in which normal colonocytes and stroma cells were stained. If no immunostaining of normal tissue could be demonstrated, the results were considered unreliable.

*MLH1 *promoter hypermethylation by Methylation Sensitive Multiplex Ligation-dependent Probe Amplification (MS-MLPA), and *BRAF *p.Val600Glu mutation by direct sequencing from tumor DNA was also assess when MLH1 loss of expression was detected.

### Statistical analysis

Hardy-Weinberg equilibrium was calculated for the control, sporadic CRC and familial CRC groups. Allelic and genotype frequencies were calculated. In the case-control study of sporadic CRC, we estimated the odds ratio (OR) and 95% confidence interval (95% CI) for the p.Lys618Ala variant using unconditional logistic regression adjusted for age and sex. We analysed for potential effect modification by age using an analysis stratified according to median age at diagnosis for the sporadic CRC cases (≤70 years or >70 years). A χ^2 ^test was used to evaluate differences in p.Lys618Ala carrier frequencies between the tumour and control groups. A probability level of <0.05 was considered significant.

## Results

No discordances were detected in the genotyping quality control. The genotype distributions in the control, sporadic and familial CRC populations did not deviate significantly from that expected for a population in Hardy-Weinberg equilibrium (Table 1).

**Table 1:** Allelic and genotypic frequencies and Hardy-Weinberg equilibrium

	Controls n = 411	Sporadic CRC n = 373	Familial CRC* n = 250	Total n = 1034
**Allele frequencies**				
**AA** **GC**	0.9866 0.0134	0.9879 0.0121	0.9860 0.0140	0.9869 0.0131

**Genotype frequencies**				
**AA/AA** **AA/GC** **GC/GC**	0.9732 0.0268 0	0.9759 0.0241 0	0.9720 0.0280 0	0.9739 0.0261 0

**HW equilibrium (p)**	0.9639	0.9706	0.9698	0.9448

*Individuals from apparently unrelated families

**Figure 1:**
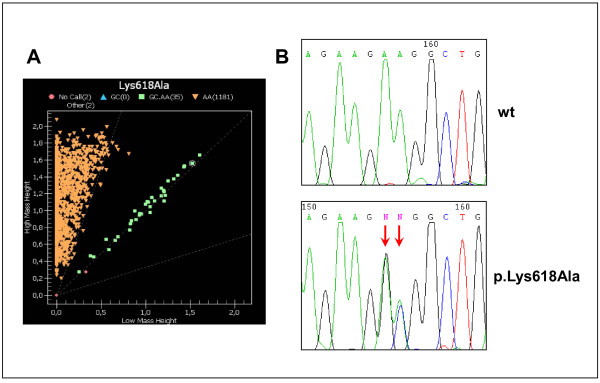
Results of genotyping for the p.Lys618Ala variant using the iPLEX Sequenom (A) and sequencing (B) methods.

Twenty-seven individuals were heterozygous for the p.Lys618Ala variant (Figure 1); 11 were controls (11/411, 2.68%), nine were CRC patients from the sporadic group (9/373, 2.41%) and seven were CRC patients from the familial group (7/250, 2.8%). None of the individuals was homozygous for the minor allele.

There were no significant associations in the case-control and case-case studies (80% detection power, OR = 3.0; two-sided test, alpha level = 5%) (Table 2) and no statistically significant associations when the OR was adjusted for age and sex.

**Table 2:** Results of case-control and case-case analyses. Odds ratios (ORs) and 95% confidence intervals (95% CIs) for the p.Lys618Ala variant

	Sporadic vs Controls	Familial vs Controls	Sporadic vs Familial
**OR [95% CI ]**	0.899 [0.378-2.139]	1.048 [0.414-2.655]	0.955 [0.377-2.417]

**p value**	1	1	1

**Figure 2:**
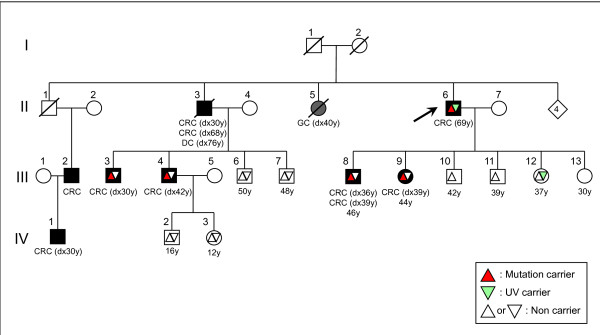
Pedigree for Family #1 (CRC: Colorectal cancer; GC: Gastric cancer; DC: Duodenal cancer).

In one of the families with LS, the index subject was heterozygous for a pathogenic *MLH1 *variant (c.767C>T; p.Arg226X) and the p.Lys618Ala variant. Two of his offspring, who were diagnosed with CRC at the ages of 36 and 39 years, carried the deleterious variant but not the p.Lys618Ala variant. An unaffected daughter (III-12) carried the p.Lys618Ala variant but not the deleterious variant. Two nephews (III-3; III-4) were also diagnosed with CRC at the ages of 30 and 42 years and they carried only the deleterious variant. Two other healthy nephews (III-6; III-7) had the wild types of the two variants (Family #1, Figure 2).

**Figure 3:**
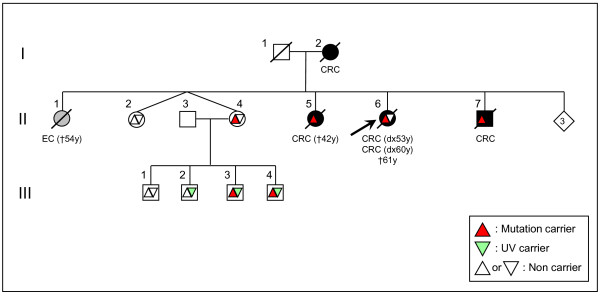
Pedigree for Family #2 (CRC: Colorectal cancer; EC: Endometrial cancer).

In the second LS family, the index subject, one sister and one brother with CRC (II-5; II-6; II-7, respectively) had a deleterious variant in *MSH6 *(c.3013C>T; p.Arg1005X) but did not have the p.Lys618Ala variant. This variant was present in only three of four unaffected nephews (III-2; III-3; III-4) and was inherited from the parental branch, in which there was no familial history of cancer. Individuals III-3 and III-4 inherited also the deleterious variant. No genetic testing was available from the father or paternal relatives (Family #2, Figure 3).

**Figure 4:**
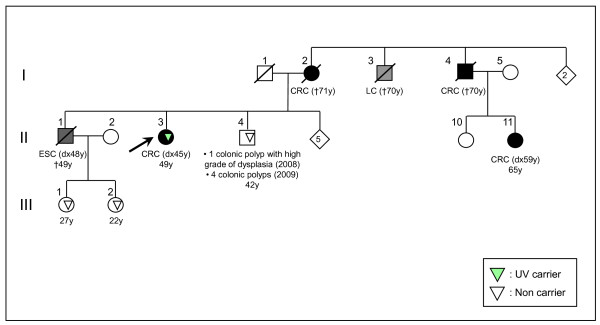
Pedigree for Family #3 (CRC: Colorectal cancer; ESC: Oesophageal cancer; LC: Lung cancer).

The p.Lys618Ala variant was present in the third family that fulfilled the Amsterdam II Criteria. A first-grade familiar non-carrier of this variant was diagnosed with a colonic polyp with a high grade of dysplasia at the age of 39 years and with four colonic polyps at the age of 42 years (Family #3, Figure 4).

Of the 17 CRC patients with the Lys618Ala variant, two had MSI (11.8%), one in the familial CRC group (1/8) and one in the sporadic CRC group (1/9).

The MSI-positive patient from the familial CRC group showed loss of immunohistochemical expression of MLH1. This is the index subject (II-3) for the third family (Figure 4) and no hypermethylation of *MLH1 *gene promoter; no *BRAF *p.Val600Glu mutation were detected in this case.

## Discussion

The accelerated development of genetic counselling in cancer during the past few years is due to the feedback and interactive information sharing on genetic studies, clinical management and psychological issues in families with a high risk of cancer. Identification of deleterious variants in such families is essential for accurate assessment of individual risk and, if required, subsequent inclusion into a personalized surveillance programme.

Unfortunately, genetic testing for hereditary cancer frequently fails to identify unambiguous deleterious variants. Erroneous classification of a genetic variant may have a great effect on at-risk familial who undergo genetic testing for risk prediction because it results in incorrect clinical recommendations.

**Figure 5:**
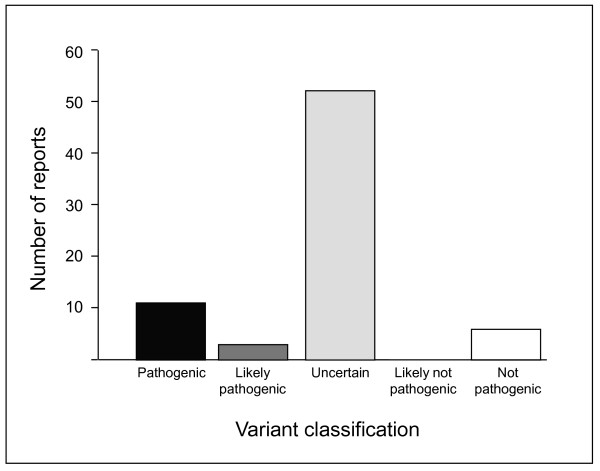
Classification of the MLH1 p.Lys618Ala variant according to the InSiGHT database (accessed on 07/2010).

LS is the most common hereditary CRC-predisposing syndrome and accounts for 3% of unselected CRC cases. A significant proportion of DNA variations found in patients suspected of having LS are UVs (32%, 18% and 38% for *MLH1*, *MSH2 *and *MSH6*, respectively) [6]. The pathogenicity of the MLH1 p.Lys618Ala variant remains controversial because of conflicting data [InSiGHT, http://www.insight-group.org] (Figure 5).

The p.Lys618Ala substitution replaces a charged amino acid with a neutral one, and occurs alongside four charged amino acids that are well conserved in mammals. *In silico *predictions of the pathogenicity of this variant using the PolyPhen http://genetics.bwh.harvard.edu/pph/ and SIFT http://sift.jcvi.org/ computational program were discordant; the SIFT analysis classified it as a tolerant variant and the PolyPhen analysis classed it as possibly damaging [9]. It has been shown that this variant may reduce the binding ability of MLH1 to PMS2 in HCT116 cells co-transfected with mutated *MLH1 *and wild-type *PMS2 *[10]. In contrast, it had no effect on the ability of MLH1 to bind PMS2 in a co-immunoprecipitation assay [9]. Functional analysis using the pCAS *ex vivo *splicing assay and RNA analysis also demonstrated no effect [11]. Moreover, a significant decrease in MLH1 protein stability has been found for the p.Lys618Ala variant [9].

The results of *in silico *prediction and functional assays alone are insufficient to determine whether this variant is deleterious or a rare functional polymorphism. For this purpose, it is necessary to integrate indirect evidence with direct genetic evidence involving clinical observations of disease occurrence.

The frequency of variants in unaffected controls is used often to distinguish between neutral and potentially deleterious variants. If the frequency of a variant among a few hundred controls is ≥1%, it is highly unlikely to be a high-risk variant. In such cases, it is still possible that the variant will be associated with a modest risk of the disease [3]. Case-control studies enable quantification of the disease risk associated with the variant. The main disadvantage of such analyses is that a large sample size is required to obtain sufficient power to detect the lower risk level. The sample size required is related inversely to the frequency of the variant in the population. The sample size used in the present study resulted in 80% power to detect an OR of 3.0 (two-sided test; alpha level, 5%). The frequency of the p.Lys618Ala variant in our control series was 2.7% and no significant differences were observed in the sporadic and familial groups, indicating that a high penetrance effect for colorectal carcinogenesis can be excluded. Similar results were reported in case-control studies on Scottish and Danish populations [12,13].

As most disease pedigrees are small, it is difficult to obtain a sufficient number of samples from affected and informative unaffected individuals. Moreover, LS deleterious variants are not completely penetrant. For these reasons, it is rarely possible to categorize variants as deleterious based on segregation alone. The co-occurrence of another known deleterious variant reduces the likelihood that an UV is truly deleterious, especially when both variants are located in *trans *[3]. To our knowledge, co-occurrence of a deleterious variant in one of the LS genes with the p.Lys618Ala variant has been observed in only two families. Liu et al [14] described an index subject from a LS family with two heterozygous variants (c.546-2A>G and c.1852_1853AA>GC); only the former segregated with LS in the family. Similarly, Steinke et al [15], described the co-occurrence of the p.Lys618Ala (c.1852_1853AA>GC) variant with the MSH6 p.Arg1068X (c.3202C>T) deleterious variant.

Herein, we describe the coexistence of the p.Lys618Ala variant with deleterious variants in another two unrelated LS families. In one family, the allele distribution of the pathogenic and unclassified variant was in *trans*, in the other family the pathogenic variant was detected in the MSH6 gene and only the deleterious variant co-segregated with the disease in both families. This evidence indicates that the p.Lys618Ala variant is not deleterious.

The molecular hallmark of LS tumours is an MSI phenotype, a functional consequence of MMR deficiency. It is expected that the putative germ-line mutation responsible for LS would confer the MSI phenotype. We tested the MSI status of 17 tumours from p.Lys618Ala carriers and detected only two cases of MSI (11.8%). Taking into consideration the bias caused by the over-representation of Bethesda Criteria-positive tumours in this subset of cases (8/17), the MSI frequency was not significantly different from that in the unselected CRC group [7]. This is further proof that the presence of this variant is irrelevant to the functional inactivation of *MLH1 *in CRC patients.

Nonetheless, we cannot exclude the possibility that this variant may result in a small increase in susceptibility to CRC or adenomas, as was suggested by Fearnhead et al [16]. Further studies with appropriate sample sizes are required to address the low penetrance effect of this variant in CRC.

Finally, we hypothesize that the clinical significance of a genetic variant may differ according to genetic background. Gene functionality may be the net result of the effects of allelic structures and their interactions with environmental factors. It is possible that low-penetrance variants behave differently in different populations, making it difficult to make predictions in terms of conferred risk.

## Conclusions

Taken together, the results of this study and others indicate that the c.1852_1853AA>GC variant should be considered a neutral variant for LS. These findings have considerable relevance for the clinical management of CRC probands and their relatives.

## Competing interests

The authors declare that they have no competing interests.

## Authors' contributions

ACastillejo participated in the design and coordination of the study, in the molecular genetic studies and helped to draft the manuscript. CG, AMC, VMB, CE, MIC, LPC, CRP and ACarracedo participated in the molecular genetic studies. ABSH, AS, LB, MA and ACastells participated in the design of the study, performed the statistical analysis and helped to draft the manuscript. EO, RL, XL, JC and RJ participated in the biobanking of samples and helped to draft the manuscript. CA and AP participated in the pathological analysis of the samples and helped to draft the manuscript. JLS conceived the study, participated in its design and coordination and drafted the manuscript. All authors read and approved the final manuscript.

## Pre-publication history

The pre-publication history for this paper can be accessed here:

http://www.biomedcentral.com/1471-2350/12/12/prepub
